# 2,5-Dibenzoyl­benzene-1,4-diaminium dichloride

**DOI:** 10.1107/S1600536807068730

**Published:** 2008-01-09

**Authors:** Shui-Ping Deng, Shan Liu, Fei Yang, Ji-Ling Xu, Hong-Jun Zhu

**Affiliations:** a Department of Applied Chemistry, College of Science, Nanjing University of Technology, Nanjing 210009, People’s Republic of China

## Abstract

The asymmetric unit of the title compound, C_20_H_18_N_2_O_2_^2+^·2Cl^−^, is composed of one-half of the 2,5-dibenzoyl­benzene-1,4-diaminium dication, located on a centre of inversion, and one Cl^−^ ion. The dihedral angle between the central benzene ring and the benzoyl phenyl ring is 53.3 (2)°. In the crystal structure, ions are linked to form a two-dimensional network parallel to the (10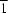) plane by N—H⋯Cl hydrogen bonds.

## Related literature

For bond-length data, see: Allen *et al.* (1987 ▶). For general background, see: Antoniadis *et al.* (1994 ▶); Imai *et al.* (1975 ▶); Kolosov *et al.* (2002 ▶); Tonzola *et al.* (2003 ▶).
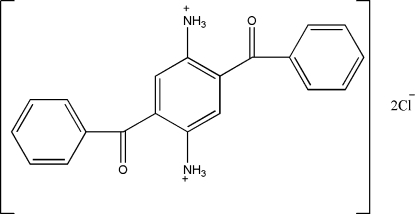


## Experimental

### 

#### Crystal data

C_20_H_18_N_2_O_2_^2+^·2Cl^−^
*M* *_r_* = 389.26
Monoclinic, 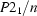

*a* = 12.373 (3) Å
*b* = 5.195 (1) Å
*c* = 14.315 (3) Å
β = 104.46 (3)°
*V* = 891.0 (4) Å^3^
*Z* = 2
Mo *K*α radiation
μ = 0.38 mm^−1^
*T* = 298 (2) K
0.40 × 0.10 × 0.10 mm

#### Data collection

Enraf–Nonius CAD-4 diffractometer
Absorption correction: ψ scan (North *et al.*, 1968 ▶) *T*_min_ = 0.862, *T*_max_ = 0.963
1754 measured reflections
1754 independent reflections
1232 reflections with *I* > 2σ(*I*)
3 standard reflections every 200 reflections intensity decay: none

#### Refinement

*R*[*F* ^2^ > 2σ(*F* ^2^)] = 0.055
*wR*(*F* ^2^) = 0.162
*S* = 1.08
1754 reflections
130 parameters
3 restraints
H atoms treated by a mixture of independent and constrained refinement
Δρ_max_ = 0.30 e Å^−3^
Δρ_min_ = −0.28 e Å^−3^

### 

Data collection: *CAD-4 Software* (Enraf–Nonius, 1985 ▶); cell refinement: *CAD-4 Software*; data reduction: *XCAD4* (Harms & Wocadlo, 1995 ▶); program(s) used to solve structure: *SHELXS97* (Sheldrick, 2008 ▶); program(s) used to refine structure: *SHELXL97* (Sheldrick, 2008 ▶); molecular graphics: *SHELXTL* (Bruker, 2000 ▶); software used to prepare material for publication: *SHELXTL*.

## Supplementary Material

Crystal structure: contains datablocks I, global. DOI: 10.1107/S1600536807068730/ci2546sup1.cif

Structure factors: contains datablocks I. DOI: 10.1107/S1600536807068730/ci2546Isup2.hkl

Additional supplementary materials:  crystallographic information; 3D view; checkCIF report

## Figures and Tables

**Table 1:** Hydrogen-bond geometry (Å, °)

*D*—H⋯*A*	*D*—H	H⋯*A*	*D*⋯*A*	*D*—H⋯*A*
N1—H1N⋯Cl1^i^	0.87 (4)	2.29 (4)	3.155 (3)	172 (4)
N1—H2N⋯Cl1	0.87 (4)	2.33 (4)	3.187 (3)	174 (4)
N1—H3N⋯Cl1^ii^	0.87 (3)	2.29 (3)	3.159 (4)	175 (2)

Symmetry codes: (i) 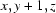; (ii) 
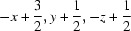
.

**Fig. 1.**
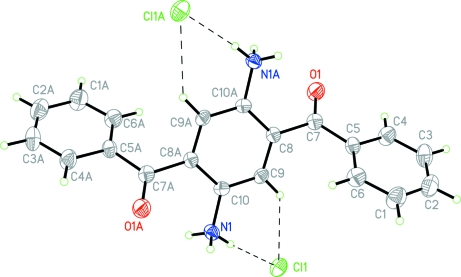
The molecular structure of the title compound, showing 40% probability displacement ellipsoids. Atoms labelled with the suffix a are generated by the symmetry operations (1 - x, 1 - y, -z). Hydrogen bonds are shown as dashed lines.

**Fig. 2.**
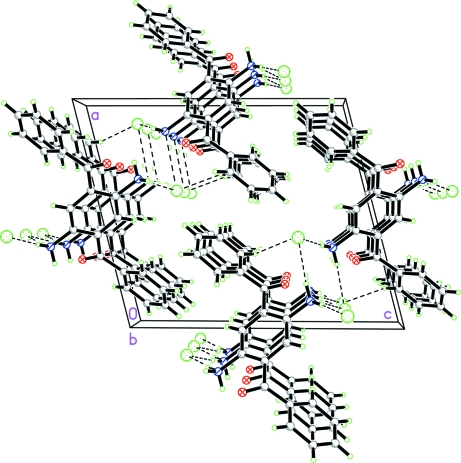
Crystal packing of the title compound. Hydrogen bonds are shown as dashed lines.

## supplementary crystallographic information

### Comment

2,5-Dibenzoyl-1,4-phenylenediamine (DBPDA) is one of the important monomers, being utilized to synthesize organic semiconductors and conjugated polymers containing anthrazoline unit (Tonzola *et al.*, 2003), which are of wide current interest for applications in electronic and optoelectronic devices including light-emitting diodes (Kolosov *et al.*, 2002), thin film transistors, and photovoltaic cells (Antoniadis *et al.*, 1994). We report here the crystal structure of the title compound.

The asymmetric unit is composed of one-half of the 2,5-dibenzoyl-1,4-phenylenediaminium dication located on a centre of inversion, and one chloride ion (Fig.1). The bond lengths and angles are within normal ranges (Allen *et al.*, 1987). The dihedral angle between the C1—C6 and C8—C10/C8A—C10A rings is 53.3 (2)°.

In the crystal structure, molecules are connected together by N—H···Cl hydrogen bonds (Table 1) to form a two-dimensional network parallel to the (1 0 1) plane (Fig. 2).

### Experimental

2,5-Dibenzoyl-1,4-phenylenediamine was synthesized as reported elsewhere (Imai *et al.*, 1975). Single crystals suitable for X-ray diffraction were obtained by dissolving the compound (2.0 g, 6.3 mmol) in hydrochloric acid (50 ml, 1.0 mol/l) and allowing the solution to evaporate at room temperature for about 25 d.

### Refinement

N-bound H atoms were located in a difference map and refined with the N—H distances restrained to be equal. C-bound H atoms were positioned geometrically (C—H = 0.93 Å) and constrained to ride on their parent atoms, with *U*_iso_(H) = 1.2*U*_eq_(C).

### Crystal data

**Table:**

C_20_H_18_N_2_O_2_^2+^·2Cl^–^	*F*_000_ = 404
*M**_r_* = 389.26	*D*_x_ = 1.451 Mg m^−^^3^
Monoclinic, *P*2_1_/*n*	Mo *K*α radiation λ = 0.71073 Å
Hall symbol: -P 2yn	Cell parameters from 25 reflections
*a* = 12.373 (3) Å	θ = 9–13º
*b* = 5.195 (1) Å	µ = 0.38 mm^−^^1^
*c* = 14.315 (3) Å	*T* = 298 (2) K
β = 104.46 (3)º	Block, colourless
*V* = 891.0 (4) Å^3^	0.40 × 0.10 × 0.10 mm
*Z* = 2	

### Data collection

**Table:**

Enraf–Nonius CAD-4 diffractometer	1232 reflections with *I* > 2σ(*I*)
Radiation source: fine-focus sealed tube	*R*_int_ = 0.0000
Monochromator: graphite	θ_max_ = 26.0º
*T* = 298(2) K	θ_min_ = 2.0º
ω/2θ scans	*h* = −15→14
Absorption correction: ψ scan(North *et al.*, 1968)	*k* = 0→6
*T*_min_ = 0.862, *T*_max_ = 0.963	*l* = 0→17
1754 measured reflections	3 standard reflections
1754 independent reflections	every 200 reflections

### Refinement

**Table:**

Refinement on *F*^2^	Secondary atom site location: difference Fourier map
Least-squares matrix: full	Hydrogen site location: inferred from neighbouring sites
*R*[*F*^2^ > 2σ(*F*^2^)] = 0.055	H atoms treated by a mixture of independent and constrained refinement
*wR*(*F*^2^) = 0.162	*w* = 1/[σ^2^(*F*_o_^2^) + (0.0704*P*)^2^ + 0.5685*P*] where *P* = (*F*_o_^2^ + 2*F*_c_^2^)/3
*S* = 1.08	(Δ/σ)_max_ = 0.001
1754 reflections	Δρ_max_ = 0.31 e Å^−^^3^
130 parameters	Δρ_min_ = −0.27 e Å^−^^3^
3 restraints	Extinction correction: none
Primary atom site location: structure-invariant direct methods	

### Special details

**Table:**

Geometry. All e.s.d.'s (except the e.s.d. in the dihedral angle between two l.s. planes) are estimated using the full covariance matrix. The cell e.s.d.'s are taken into account individually in the estimation of e.s.d.'s in distances, angles and torsion angles; correlations between e.s.d.'s in cell parameters are only used when they are defined by crystal symmetry. An approximate (isotropic) treatment of cell e.s.d.'s is used for estimating e.s.d.'s involving l.s. planes.
Refinement. Refinement of *F*^2^ against ALL reflections. The weighted *R*-factor *wR* and goodness of fit *S* are based on *F*^2^, conventional *R*-factors *R* are based on *F*, with *F* set to zero for negative *F*^2^. The threshold expression of *F*^2^ > σ(*F*^2^) is used only for calculating *R*-factors(gt) *etc*. and is not relevant to the choice of reflections for refinement. *R*-factors based on *F*^2^ are statistically about twice as large as those based on *F*, and *R*- factors based on ALL data will be even larger.

### Fractional atomic coordinates and isotropic or equivalent isotropic displacement parameters (Å^2^)

**Table:**

	*x*	*y*	*z*	*U*_iso_*/*U*_eq_	
O1	0.2971 (2)	0.0151 (5)	−0.11097 (19)	0.0386 (7)	
N1	0.6436 (3)	0.5247 (6)	0.1879 (2)	0.0265 (7)	
H1N	0.635 (4)	0.655 (7)	0.224 (3)	0.073 (17)*	
H2N	0.633 (3)	0.394 (7)	0.222 (3)	0.045 (12)*	
H3N	0.714 (2)	0.517 (8)	0.188 (3)	0.040 (12)*	
C1	0.1700 (4)	0.2318 (8)	0.1666 (3)	0.0438 (10)	
H1	0.1688	0.3459	0.2164	0.053*	
C2	0.1003 (3)	0.0237 (9)	0.1510 (3)	0.0450 (10)	
H2	0.0508	−0.0008	0.1896	0.054*	
C3	0.1022 (3)	−0.1489 (8)	0.0793 (3)	0.0442 (10)	
H3	0.0558	−0.2922	0.0706	0.053*	
C4	0.1732 (3)	−0.1104 (7)	0.0200 (3)	0.0372 (9)	
H4	0.1731	−0.2260	−0.0296	0.045*	
C5	0.2448 (3)	0.1002 (7)	0.0339 (3)	0.0274 (8)	
C6	0.2430 (3)	0.2733 (7)	0.1081 (3)	0.0352 (9)	
H6	0.2902	0.4154	0.1184	0.042*	
C7	0.3141 (3)	0.1395 (6)	−0.0361 (2)	0.0262 (8)	
C8	0.4074 (3)	0.3311 (6)	−0.0166 (2)	0.0230 (7)	
C9	0.4812 (3)	0.3497 (7)	0.0737 (2)	0.0250 (7)	
H9	0.4697	0.2484	0.1239	0.030*	
C10	0.5707 (3)	0.5142 (6)	0.0907 (2)	0.0230 (7)	
Cl1	0.59773 (8)	0.02097 (17)	0.29914 (7)	0.0350 (3)	

### Atomic displacement parameters (Å^2^)

**Table:**

	*U*^11^	*U*^22^	*U*^33^	*U*^12^	*U*^13^	*U*^23^
O1	0.0478 (16)	0.0345 (15)	0.0357 (14)	−0.0127 (13)	0.0148 (12)	−0.0065 (12)
N1	0.0268 (15)	0.0226 (16)	0.0272 (15)	−0.0029 (14)	0.0015 (12)	0.0026 (14)
C1	0.054 (3)	0.037 (2)	0.047 (2)	−0.004 (2)	0.025 (2)	−0.008 (2)
C2	0.039 (2)	0.051 (3)	0.050 (2)	0.002 (2)	0.0205 (19)	0.010 (2)
C3	0.041 (2)	0.035 (2)	0.060 (3)	−0.0108 (19)	0.019 (2)	0.004 (2)
C4	0.048 (2)	0.026 (2)	0.040 (2)	−0.0095 (18)	0.0165 (18)	−0.0018 (17)
C5	0.0274 (18)	0.0209 (17)	0.0343 (19)	−0.0024 (14)	0.0082 (15)	0.0009 (15)
C6	0.042 (2)	0.0258 (19)	0.039 (2)	−0.0010 (16)	0.0108 (17)	0.0012 (16)
C7	0.0291 (18)	0.0184 (16)	0.0309 (18)	0.0025 (14)	0.0070 (15)	0.0041 (14)
C8	0.0242 (16)	0.0171 (16)	0.0276 (17)	0.0007 (13)	0.0059 (13)	0.0017 (14)
C9	0.0307 (18)	0.0199 (17)	0.0254 (17)	−0.0008 (14)	0.0091 (14)	0.0050 (14)
C10	0.0284 (17)	0.0182 (16)	0.0214 (15)	0.0012 (14)	0.0046 (13)	−0.0003 (14)
Cl1	0.0418 (5)	0.0276 (5)	0.0393 (5)	0.0038 (4)	0.0168 (4)	0.0054 (4)

### Geometric parameters (Å, °)

**Table:**

O1—C7	1.223 (4)	C4—C5	1.390 (5)
N1—C10	1.458 (4)	C4—H4	0.93
N1—H1N	0.87 (3)	C5—C6	1.396 (5)
N1—H2N	0.87 (3)	C5—C7	1.487 (5)
N1—H3N	0.87 (2)	C6—H6	0.93
C1—C2	1.366 (6)	C7—C8	1.497 (5)
C1—C6	1.393 (5)	C8—C9	1.388 (5)
C1—H1	0.93	C8—C10^i^	1.409 (4)
C2—C3	1.367 (6)	C9—C10	1.372 (5)
C2—H2	0.93	C9—H9	0.93
C3—C4	1.381 (5)	C10—C8^i^	1.409 (4)
C3—H3	0.93		
			
C10—N1—H1N	117 (3)	C4—C5—C6	119.0 (3)
C10—N1—H2N	111 (3)	C4—C5—C7	117.7 (3)
H1N—N1—H2N	102 (4)	C6—C5—C7	123.1 (3)
C10—N1—H3N	112 (3)	C1—C6—C5	119.6 (4)
H1N—N1—H3N	108 (4)	C1—C6—H6	120.2
H2N—N1—H3N	105 (4)	C5—C6—H6	120.2
C2—C1—C6	120.1 (4)	O1—C7—C5	121.1 (3)
C2—C1—H1	119.9	O1—C7—C8	118.1 (3)
C6—C1—H1	119.9	C5—C7—C8	120.9 (3)
C1—C2—C3	120.8 (4)	C9—C8—C10^i^	117.1 (3)
C1—C2—H2	119.6	C9—C8—C7	121.2 (3)
C3—C2—H2	119.6	C10^i^—C8—C7	121.6 (3)
C2—C3—C4	120.0 (4)	C10—C9—C8	121.5 (3)
C2—C3—H3	120.0	C10—C9—H9	119.2
C4—C3—H3	120.0	C8—C9—H9	119.2
C3—C4—C5	120.4 (4)	C9—C10—C8^i^	121.4 (3)
C3—C4—H4	119.8	C9—C10—N1	118.1 (3)
C5—C4—H4	119.8	C8^i^—C10—N1	120.5 (3)

Symmetry codes: (i) −*x*+1, −*y*+1, −*z*.

### Hydrogen-bond geometry (Å, °)

**Table:**

*D*—H···*A*	*D*—H	H···*A*	*D*···*A*	*D*—H···*A*
N1—H1N···Cl1^ii^	0.87 (4)	2.29 (4)	3.155 (3)	172 (4)
N1—H2N···Cl1	0.87 (4)	2.33 (4)	3.187 (3)	174 (4)
N1—H3N···Cl1^iii^	0.87 (3)	2.29 (3)	3.159 (4)	175 (2)

Symmetry codes: (ii) *x*, *y*+1, *z*; (iii) −*x*+3/2, *y*+1/2, −*z*+1/2.
